# Ameliorating Effects of Exogenously Applied Proline on Seed Composition, Seed Oil Quality and Oil Antioxidant Activity of Maize (*Zea mays* L.) under Drought Stress

**DOI:** 10.3390/ijms14010818

**Published:** 2013-01-04

**Authors:** Qasim Ali, Farooq Anwar, Muhammad Ashraf, Nazamid Saari, Rashida Perveen

**Affiliations:** 1 Department of Botany, Government College University, Faisalabad-38040, Pakistan; 2 Department of Chemistry, University of Sargodha, Sargodha-40100, Pakistan; 3 Department of Botany, University of Agriculture, Faisalabad-38040, Pakistan; 4 Faculty of Food Science and Technology, Universiti Putra Malaysia, UPM-43400 Serdang, Selangor, Malaysia; 5 Department of Physics, University of Agriculture, Faisalabad-38040, Pakistan

**Keywords:** seed composition, oil attributes, fatty acids, antioxidant activity, lipophilic minor components

## Abstract

This study was carried out to appraise whether or not the exogenous application of a potential osmoprotectant, proline, could ameliorate the adverse effects of drought stress on maize seed and seed oil composition, as well as oil antioxidant activity. Water stress reduced the kernel sugar, oil, protein and moisture contents and most of the seed macro- and micro-elements analyzed in both maize cultivars but it increased the contents of seed fiber and ash. Water stress increased the oil oleic acid content with a subsequent decrease in the amount of linoleic acid, resulting in an increased oil oleic/linoleic ratio for both maize cultivars. However, no variation was observed in oil stearic and palmitic acids content due to water stress. A considerable drought induced an increase in seed oil α-, γ-, δ- and total tocopherols and flavonoids were observed in both maize cultivars. However, oil phenolic and carotenoid content as well as 1,1-diphenyl-2-picryl-hydrazyl (DPPH) free radical scavenging activity decreased. Foliar-applied proline significantly increased the content of seed sugar, oil, protein, moisture, fiber and ash in both maize cultivars under well irrigated and water deficit conditions. Furthermore, exogenous application of proline increased the oil oleic and linoleic acid contents. The concentrations of antioxidant compounds namely phenolics, carotenoids, flavonoids and tocopherols estimated in the seed oil increased due to foliar-applied proline under water deficit conditions that was positively correlated with the enhanced oil DPPH free radical scavenging activity. Moreover, the increase in the contents of these antioxidant compounds and oil antioxidant activity due to the foliar application of proline was noted to be more pronounced under water deficit conditions.

## 1. Introduction

It is now well known that different environmental factors not only influence the growth and productivity but also significantly affect the nutrients profile of oil-seed crops [ 1]. Of the different unfavorable environmental conditions, a shortage of water is considered to be one of the most important factors that can alter the yield and quality of seeds as well as the physicochemical attributes of the related oils in the oil-seed crops [ 2, 3].

A large proportion of world food demand is fulfilled by cereal crops [ 4]. Cereal grains are predominantly composed of carbohydrates, mostly in the form of starch, along with considerable amounts of protein and lipids, vitamins, and minerals [ 5, 6]. Among different cultivated economically important cereal crops, maize (*Zea mays* L.) not only has sufficient amount of carotenoids, tocopherols and oil, but also has significant contents of starch and protein compared with other major food crops such as rice and wheat. Although maize is principally cultivated for carbohydrate production, in the past several years, it has gained great significance as a source of vegetable oil for the food industry.

Among the common edible oils, maize oil is considered the best due to its large amount of unsaturated fatty acids (oleic and linoleic acids), which are in the range from 65% to 85% depending upon the type of cultivar and the environmental conditions to which a cultivar is exposed [ 6, 7]. The oil is also a rich source of secondary metabolic antioxidant compounds, such as phenolics, flavonoids, carotenoids and tocopherols [ 3, 6]. These compounds have a significant role in oil oxidative stability. Although these compounds are considered as non-nutritive, their antioxidative and bioactive properties have made them indispensable for human health [ 7]. They possess multiple biological effects such as anti-artherogenic, anti-inflammatory, antiallergenic, antimicrobial, antithrombotic and cardioprotective [ 8].

Various organic compatible solutes that effectively take part in plant stress tolerance include proline (PRO), glycine betaine (GB), trehalose (Tre) and several others [ 6, 9–11]. Of these organic osmolytes, proline (an imino acid) is also found widely in plants and accumulates in large quantities in response to environmental stresses such as drought, salinity, extreme of temperature, *etc*. [ 12–15]. It actively takes part in plant osmotic adjustment under stressful environmental conditions [ 16]. In addition to its role as an osmolyte for osmotic adjustment, it actively takes part to stabilize sub-cellular structures, biological membranes, proteins, and scavenge free radicals. It also plays a vital role in buffering cellular redox potential under stressful environmental conditions. Normally, proline accumulation in plants, is in response to drought or salinity stress occurs in the cytosol where it contributes substantially to the cytoplasmic osmotic adjustment [ 15–17].

There are many reports that depict that exogenous application of proline as foliar spray can play an important role in enhancing plant tolerance against abiotic stresses. This ameliorationg effect of exogenously applied proline can be in the form of either osmoprotection [ 18, 19] or cryoprotection or protection against reactive oxygen species [ 20, 21]. For example, in various plant species growing under stress conditions, exogenously supplied proline provided osmoprotection and facilitated plant growth [9, 19, 22, 23]. Proline can also protect cellular and subcellular membranes from oxidative stress by enhancing the activities of various antioxidant enzymes including superoxide dismutase (SOD), peroxidase (POD) and catalase (CAT) [ 24, 25]. Such protective effects of exogenously applied proline were indicated to be due to plasma membrane stabilization [ 26,27]. Although exogenous application of proline as foliar spray has been employed to enhance stress tolerance in a number of crops [ 19, 23], very little information is available in the literature on the effects of foliar application of this osmolyte in altering the seed composition, levels of different fatty acids, tocopherols, carotenoids, phenolics and flavonoids particularly in the seed oil of maize. In view of this, it was hypothesized that exogenous application of proline as foliar spray improves the seed composition in terms of quantity as well as quality of seed oil in maize grown under water deficit conditions. Thus, the premier objective of conducting the present study was to assess up to what extent exogenous application of proline could alter seed composition, oil yield, and fatty acids, tocopherols, antioxidants and physico-chemical attributes of the seed oil derived from maize plants grown under water deficit conditions.

## 2. Results and Discussion

Grain yield and its quality are governed by a number of factors particularly the duration and rate of grain filling [ 28] and availability of assimilates, that are negatively influenced under water deficit conditions [ 1–3, 6, 29]. The maize seed proximate analysis in the present study depicted that imposition of drought stress reduced the seed sugar, oil, protein and moisture content and increased the fiber and ash contents of the two maize cultivars. However, seed starch contents remained un-affected due to water stress. Exogenous application of proline as foliar spray significantly increased the seed oil, protein, moisture, fiber and ash contents in both the cultivars under water stress and non-stress conditions showing the ameliorating effects of exogenously applied proline on seed proximate composition (Table 1). Furthermore, this enhancement in the seed proximate parameters due to foliar application of proline was more pronounced under water deficit conditions as compared with non-stress conditions. This ameliorating effect of exogenously applied proline on altering these seed chemical parameters might have been due to its role to maintain turgor in plants both under non-stress and water deficit conditions, thereby maintaining high photosynthetic efficiency [ 30] and as a result more allocation of assimilates to developing seeds [ 31].

Analysis of the quality-oriented physico-chemical attributes showed that the oil saponification value decreased, while oil un-saponifiable matter increased due to water stress in both maize cultivars, but oil iodine value remained unchanged. Foliar-applied proline significantly increased the oil saponification and iodine values in both cultivars, but the oil un-saponifiable matter decreased due to exogenously applied proline both under non-stress and water stress conditions. Of the other seed oil quality-oriented parameters, peroxide and *p*-anisidine values, oil density and seed oil free fatty acids increased in both maize cultivars due to desiccation stress. Exogenous application of proline as foliar spray slightly increased the oil *p*-anisidine value of both maize cultivars under normal and water deficit conditions, but, oil peroxide value and free fatty acids decreased slightly due to foliar application of proline in both maize cultivars both under water stress and non-stress conditions. Similarly, maize kernel oil dienes and trienes, a measure of the extent of oil oxidation, were also affected due to water limited conditions. A significant decrease in oil dienes with a subsequent increase in trienes was observed due to water stress. Foliar-applied proline significantly decreased the seed oil dienes and trienes of both maize cultivars both under stress and non-stress conditions (Table 2). Such improvement in seed oil quality in terms of oil physico-chemical attributes due to foliar-applied proline both under non-stress and water deficit conditions can be related to an earlier study on maize seed oil [ 6] in which it was reported that exogenous application of a potential organic osmolyte glycinebetaine showed ameliorating effects on these seed oil physico-chemical attributes under water deficit and non-stressed conditions.

**Figure 1:**
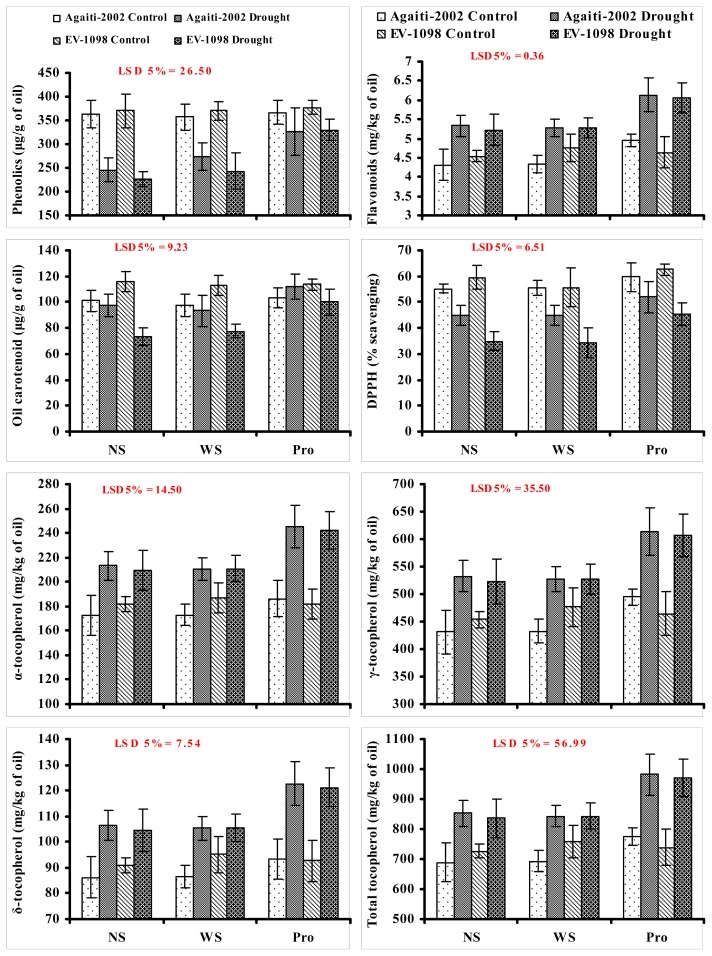
Phenolic, flavonoid, carotenoid, and tocopherol contents and 1,1-diphenyl-2- picryl-hydrazyl (DPPH) scavenging activity of seed oil of two maize (*Zea mays* L.) cultivars influenced by exogenous application of proline as foliar spray at vegetative growth stage under drought stress and non-stress conditions (*n* = 4 ± SE). NS: No spray; WS: Water spray; Pro: 30 m*M* proline.

It is generally known that different abiotic stresses including desiccation stress cause the production of highly reactive oxygen species (ROS) such as O_2_^−^, H_2_O_2_ and OH in different plant organelles such as mitochondria, peroxisomes, and chloroplasts [ 32–34]. In defense, to counteract the excess production of ROS, plants synthesize various hydrophilic (ascorbic acid, and salicylic acid) and lipophilic (tocopherols) secondary metabolic compounds that play a significant role in scavenging these ROS in various plant parts. As in most crop species, the desiccation is the final phase of seed development [ 35], so, seeds contain low amount of water as well as low levels of few water-soluble antioxidants and antioxidant enzymes [ 36]. So, at this desiccated seed filling stage they are not properly safeguarded from oxidative stress. Thus, during this seed filling stage, the amount of lipophilic antioxidant compounds especially tocopherols are more important in scavenging the ROS thereby maintaining seed oil quality. Tocopherols exist in four distinct forms such as α-, β-, γ- and δ-tocopherols. These compounds differ in the number and position of methyl substitutions in the chromanol ring. They are well recognized due to their antioxidative property in vegetable oils. Their presence in vegetative oils increases the stability of lipids against autoxidation [ 37]. In the present study, water stress significantly increased α-, γ-, δ- and total tocopherol contents in the kernel oil of both maize cultivars. Of the different tocopherols analyzed, γ-tocopherol occurred abundantly in the seed oil of both maize cultivars under non-stress (430.83 to 454.81 mg kg^−1^) and water stress conditions (522.50 to 532.87 mg kg^−1^). Foliar application of proline further increased the oil content in either of the maize cultivars under both the stress (increased from 209 to 245.4, 5.22.5 to 613.5, 104.5 to 122.7 and 836–981.6 mg kg^−1^) and non-stress conditions (increased from 172.33 to 186.20, 430.83 to 494.67, 86.17 to 93.43 and 689.33 to 774.30 mg kg^−1^, respectively for α-, γ-, δ- and total tocopherol) (Figure 1). Furthermore, an increase in tocopherols due to foliar-applied proline was noted to be more distinct under water deficit conditions as compared with non-stress conditions. These findings are similar to those of Britz and Kremer [ 38] in which they reported that drought stress caused a significant increase in seed α-tocopherols in soybean. Similarly, drought stress caused increase in tocopherols has also been reported in an *Arabidopsis* mutant (*npq1*) [ 39], *Rosmarinus officinalis* [ 40], sunflower [ 2], and maize seed oils [ 3, 6]. As described earlier [ 41], tocopherols are lipophilic antioxidants and they protect the polyunsaturated fatty acids from peroxidation [ 42] so the quantification of these components is worthwhile. In the present study, a positive association has been found between un-saturated fatty acids such as oleic and linoleinic acids, and each of α, γ, δ and total tocopherol contents. These findings are analogous to those of Kriese *et al*. [ 43] and Ali *et al*. [ 3], who found a positive correlation between some fatty acids, and γ-tocopherols and total tocopherols. Furthermore, in a recent study Ali and Ashraf [ 44] reported that exogenously applied osmolytes such as glycinebetaine could improve the contents of oil tocopherols under water deficit conditions.

Flavonoids are the plants secondary metabolic compounds, which act effectively as scavengers of oxidizing molecules including singlet oxygen and free radicals. They also play an important physiological role in plant stress tolerance [ 19, 45]. In the present study, seed oil flavonoids of both maize cultivars increased due to imposition of water stress (4.30 to 5.32 mg kg^−1^). Exogenous application of proline as a foliar spray further increased the contents of oil flavonoids of both cultivars under stress regimes, but more proline-induced increase was observed in both cultivars under water deficit conditions (increased from 4.30 to 4.94 and 5.22 to 6.33 under non-stress and water deficit conditions, respectively) (Figure 1). Such changes in these oil attributes can be correlated with an earlier study in which it was observed that total flavonoid contents increased throughout the seed development in two Korean soybean cultivars during desiccation [ 46]. Similarly, the drought-induced increase in flavonoid contents in maize kernel oil as observed in the present study may have been due to their enhanced synthesis during seed development and seed maturity [ 47]. The increase in flavonoids in kernel oil of drought stressed plants due to exogenous application of proline, might have been due to delay in maturity of plants that might have delayed the seed maturity [ 48] thereby leading to more accumulation of flavonoids in the seeds [ 49].

Phenolic compounds have been reported to be present in all vegetable oils, which are very important for the oxidative stability of the polyunsaturated fatty acids of these oils [ 50]. Furthermore, it has been reported in an earlier study that seed oil total phenolic contents are indicative of the seed oil total antioxidative activity [ 46] due to the presence of the phenolic hydrogens (as hydrogen-donating radical scavengers), which predict their antioxidant activity [ 51]. Cereal grains have been reported to have the highest level of phenolics and antioxidant activity [ 52]. In some earlier studies on olive oil [ 53], rape-seed oil [ 54] and maize kernel oil [ 3, 6], it was observed that water deficit significantly decreased the concentration of oil phenolic compounds. All these reports support the results of the present study wherein drought-induced decrease in phenolic contents (from 363.33 to 245 mg kg^−1^) was observed (Figure 1). The proline-induced increase in oil phenolic contents (increased from 226.67 to 330 μg g^−1^ under water deficit conditions) in the present study can be supported by the findings of previous studies [ 44, 55], which revealed that foliar-applied compatible solutes could enhance the levels of phenolic compounds in strawberry and maize, respectively, under water deficit conditions.

In the present study, seed oil carotenoid contents of both maize cultivars decreased (from 116 to 73.33 μg g^−1^) significantly due to the imposition of water stress. This reduction in oil carotenoid contents can be related to an earlier study on different pea cultivars, where in drought stress significantly reduced the seed total carotenoid contents of different pea cultivars, but this decrease in carotenoid contents was not uniform in pea cultivars [ 56]. Exogenous application of proline significantly enhanced the accumulation of carotenoids (increased 73.33 to 111.7 μg g^−1^) in the seed oil of both maize cultivars under water deficit conditions (Figure 1) which could have been due to the reason that lipid biosynthesis in seeds during ripening is stimulated by the exogenous application of organic osmolytes [ 44].

The 1,1-diphenyl-2-picryl-hydrazyl (DPPH) radical scavenging activity, a measure of oil total antioxidant activity, decreased (55% to 32%) significantly in both maize cultivars due to drought stress. Exogenous application of proline increased (from 55% to 62% and 35% to 52%) the seed oil antioxidant activity in both maize cultivars under stress and non-stress conditions, respectively (Figure 1). In the present study, seed oil antioxidant activity in both cultivars was positively related to oil phenolics, carotenoids, flavonoids, tocopherols and oil oleic acid contents only when proline was applied as foliar spray under both stress and non-stress conditions. The strong positive correlation between total phenolics, carotenoids and antioxidant activity as observed in the present study had already been observed in cereals [ 52] and soybean [ 46], which suggests that this increase in oil antioxidant activity is contributed by the presence of high amount of phenolic compounds. Similar positive correlation in seed oil antioxidant activity and different lipophilic antioxidant compounds under water deficit condition and due to exogenous application of organic osmolytes had already been reported in some earlier studies in maize [ 3, 6].

**Figure 2:**
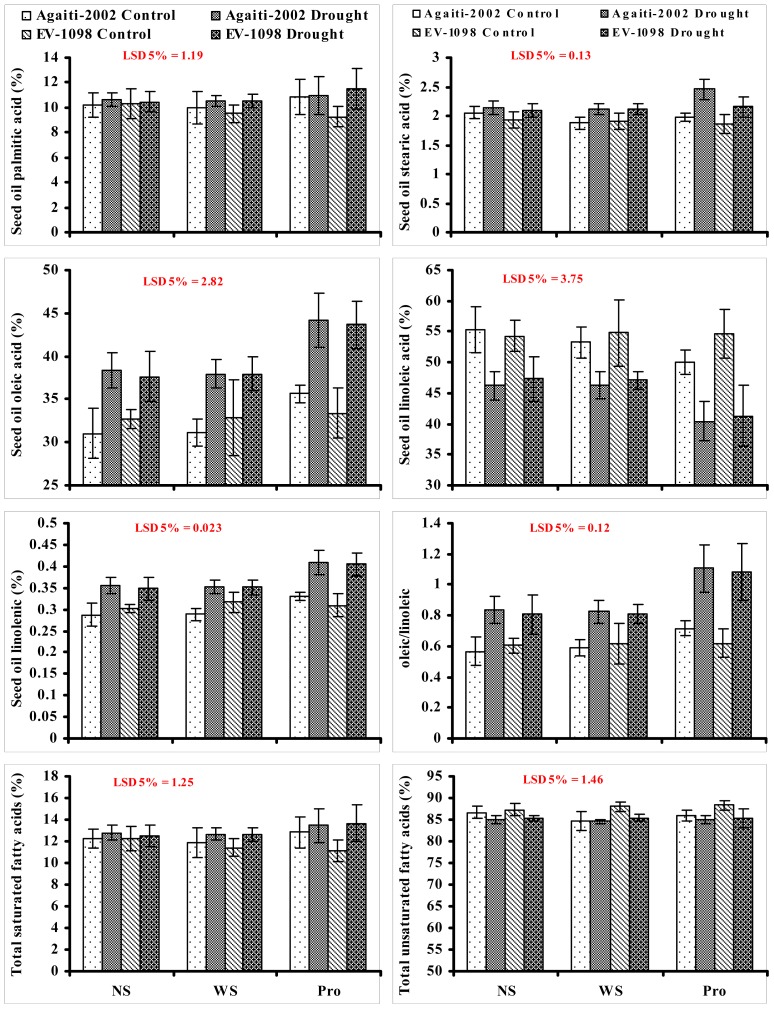
Fatty acid composition of seed oil of two maize (*Zea mays* L.) cultivars influenced by exogenous application of proline as foliar spray at vegetative growth stages under drought stress and non-stress conditions (*n* = 4 ± SE). NS = No spray; WS=Water spray; Pro=30 m*M*proline.

Due to increased industrial use of corn, the fatty acid composition of its oil is considered very vital in determination of oil nutritional quality and possible uses of oil in industrial applications. However, adverse environmental conditions such as drought have significant effects on seed oil fatty acid composition [ 2, 3, 57]. Similarly, in the present study, maize kernel oil oleic acid, linolenic acid and oleic/linoleic ratio increased, while linoleic acid decreased in both maize cultivars due to drought stress. Foliar-applied proline further increased the contents of oleic, linolenic and oleic/linoleic and decreased the linoleic acid contents. However, no significant effect of water stress or foliar-applied proline was observed on oil total saturated and unsaturated fatty acids (Figure 2). Furthermore, the increase in oil oleic acid, linolenic acid and oleic/linoleic ratio due to foliar-applied proline was more distinct in drought stressed plants. The changes in oleic/linoleic acid ratio due to exogenous application of proline under both water deficit (increased from 0.80 to 1.10) and non-stressed (increased from 0.56 to 0.71) conditions as observed in the present study shows a direct or indirect effect of proline on the enzymes involved in the biosynthesis of oleic and linoleinic acids. These results indicate that it is likely that proline had a protective effect rather than a direct involvement in fatty oil biosynthesis and storage, which occurs in liposomes or oleosomes in seeds during seed filling stage [ 35].

## 3. Experimental Section

The present study was conducted to assess the changes in composition of maize seed and seed oil derived from field grown plants sprayed with proline, one of the potential organic osmolytes. For the experimentation two maize cultivars with differential tolerance to drought Agaiti-2002 (drought tolerant) and EV-1098 (relatively drought sensitive) were used. These two cultivars are being widely used in Pakistan for maize breeding programs aiming at improving qualitative and quantitative traits of the crops. The seeds of these two maize cultivars were supplied by the Maize and Millet Research Institute, Yousaf wala (Sahiwal), Pakistan. The experiment was conducted at the research area of the New Botanical Garden, of the Department of Botany, University of Agriculture, Faisalabad, Pakistan (latitude 30°30 N, longitude 73°10 E and altitude 213 m).

The climatic conditions at the experimental site during the experimentation calculated as means were as follow: photosynthetically available radiation (*PAR*) measured at noon varied from 794 to 1154 μmol m^−2^ s^−1^, and day/night RH 33.1/75.1%, and day and night temperatures 38.3 ± 4.0 °C and 22.8 ± 3.6 °C, respectively.

The soil of the experimental site was sandy clay (average 65% clay content, 22% sand and 13% silt). The soil texture was determined by using the hygrometer method following Dewis and Freitas [ 58]. The saturation percentage of soil was 31, organic matter 0.78% and NH_4_-N 3.00, NO_3_-N 6.5, available potassium 187, phosphorous 5.6, and calcium 109 (all values of nutrients in mg kg^−1^ of dry soil). The soil electrical conductivity (EC_e_) was 2.1 dS m^−1^ and pH 8.1. Electrical conductance (ECe), pH and inorganic nutrients of the soil saturation extract were appraised following Jackson [ 59].

The whole experiment was arranged in a randomized complete block design (RCBD). On the basis of water stress treatments the main plot was divided into two sub-plots. In one sub-plot, normal irrigation was applied and in the second sub-plot, drought stress was started during the early growth stage of crop growth. Each experimental unit was replicated four times. For the preparation of soil for seed sowing, the first irrigation to both subplots was applied 15 days before sowing. When the soil was at field capacity condition, the plots were well prepared for sowing the seeds. The rate of the seed sown was 10 kg ha^−1^ of each maize cultivar that was hand-drilled by maintaining inter-row distance of 75 cm. Plants were thinned 15 days after germination to maintain plant-to-plant space 30 cm.

The first irrigation to all plots was applied 8 days after seedling emergence and thereafter, drought stress treatment was initiated by controlling irrigation schedule. After the 1st irrigation, the watering to non-stressed plants was applied regularly at 15-day-interval except the plants meant for experiencing drought stress; however, the irrigations meant for water stressed plants were applied at 21-day intervals.

Two levels of proline (Sigma Aldrich, Germany) (0 and 30 mM) were used for the experimentation, which were prepared in the surfactant tween-20 (0.1% solution) and applied foliarly only once at the vegetative stage, when the plants were at five-leaf stage. An aliquot of 500 ml of 30 mM proline solution was applied foliarly to plants in each replicate using manually operated agricultural spray equipment with an eight-liter tank capacity. The equipment tank was fitted with a brass spray lance with fine spray brass nozzle and a pump barrel made of seamless brass tube. The foliar application of proline was carried out in the evening just before sunset. Before harvest, ears were removed. The ears were dried for 3 to 4 days in air during daytime. After proper drying, kernels were separated from ears and used for further analysis. All reagents used were of analytical and HPLC grades and purchased from Sigma-Aldrich (Germany).

### 3.1. Characterization of Maize kernels and Kernel Oil

#### Proximate Analysis

Maize kernels were evaluated for different proximate analyses including crude protein, moisture, ash, crude fat, starch and crude fiber by using appropriate protocols as depicted in AACC [ 60], *i.e*., Method No. 44-15A, Method No. 08-01, Method No. 46-30, Method No. 30-25, Method 32-10, respectively.

### 3.2. Oil Extraction

For the extraction of oil, the seeds were properly dried. Then the seeds (200 g) of each treatment were crushed into 80 mesh of particle size. This accurately weighed crushed seed material was then packed in paper thimbles and placed them in a Soxhlet collector fitted with 500 mL volumetric flask. The extraction was carried out using *n*-hexane as a solvent for 8 h.

### 3.3. Chemical Parameters of Oil

Determinations of kernel oil different physico-chemical attributes including iodine value, saponification value, unsaponifiable matter, peroxide value, density and contents of free fatty acids (FFA) of the extracted oil were performed following the established AOCS methods (Cd 1-25, Cd 3-25, Ca 61-40, Cd 8-53, Cc 10a-25 and F9a-44, respectively [ 61].

Oil specific extinctions, dienes and trienes [ɛ^1%^ 1 cm (λ)] at 232 and 270 nm, as a measure of oil oxidation measure were appraised following the IUPAC method II. D. 23 [ 62]. Oil samples (0.1 mL) were diluted with iso-octane until the absorbance was in the range 0–0.8. The absorbance of the samples was read at 232 and 270 nm on a spectrophotometer (U-2001, Hitachi Instruments Inc., Tokyo, Japan). The amount of *p*-anisidine was determined following an IUPAC method II. D. 26 [ 62]. Coloured complexes of the oil samples were developed by reading the oil samples *p*-anisidine for 10 min and absorbance read at 350 nm using a spectrophotometer (U-2001, Hitachi Instruments Inc., Tokyo, Japan).

### 3.4. Fatty Acid Composition of Seed Oil

Fatty acid methyl esters (FAMEs) were prepared following the IUPAC standard method (2.301) [ 62] and quantified using a gas chromatograph (Perkin Elmer, model Clarus 500) fitted with a Rt-2340 NB (RESTEK, Corp.) methyl-lignocerate-coated (film thickness 0.20 μm) polar capillary column (60 m × 0.25 mm) and an FID detector. Analytical grade nitrogen gas with a flow rate of 5 mL/min was used as mobile phase. Other conditions maintained were: oven initial temperature, 80 °C; ramp rate, 3 °C/min; final temperature, 210 °C; detector temperature 220 °C; and injector temperature, 210 °C. The quantification of FAMEs was based on their absolute retention times after comparing with appropriate standards purchased from Sigma-Aldrich (Buchs, Switzerland).

### 3.5. Tocopherol Content

The tocopherol content in the kernel oil was determined following Lee *et al*. [ 63] and analyzed by HPLC (Sykam GmbH, Kleinostheim, Germany). The HPLC system was equipped with S-1122 dual piston solvent delivery system, and S-3210 UV/VIS diode array detector. Twenty μL of the extract were injected into the Hypersil ODS reverse phase (C18) column (5 μm particle size, 250 mm × 4.6 ID Themohypersil GmbH, (Darmstadt, Germany) fitted with a C_18_ guard column and methanol: acetonitrile: methylene chloride (50:44:6, *v*/*v*) mobile phase at 1 mL/min flow rate. The peak areas were recorded and calculated by a computer with SRI peak simple chromatography data acquisition and integration software (SRI instrument, Torrance, CA, USA) at 295 nm. The quantification of tocopherols was done by comparing the samples with pure standards purchased from Sigma-Aldrich (Buchs, Switzerland).

### 3.6. Total Phenolic Contents

The amount of total phenolics was assessed using the Folin-Ciocalteu reagent method as described by Chaovanalikit and Wrolstad [ 64]. Briefly, 10 g oil was mixed with 60 mL *n*-hexane and extracted with 60% methanol (60:40 watre:methanol *v*/*v*). The solvent was evaporated to dryness (40 °C) using a rotary evaporator (Heidolph, model, Laborota 4001, Schwabach, Germany) to obtain the crude extract. Then the crude extract (50 mg) was mixed with 0.5 mL of Folin-Ciocalteu reagent and 7.5 mL deionized water. The mixture was kept at room temperature for 10 min, and then 1.5 mL of 20% sodium carbonate (*w*/*v*) added. The mixture was heated in a water bath at 40 °C for 20 min, cooled in an ice bath and absorbance measured at 755 nm using a spectrophotometer (U-2001, Hitachi Instruments Inc. Tokyo, Japan). Amounts of TP were calculated using a gallic acid calibration curve within the range of 10–100 mg/L. The results were expressed as gallic acid equivalents (GAE) g/100 g of dry matter.

### 3.7. Total Flavonoids

Total flavonoids were appraised following the procedure of Dewanto *et al*. [ 65]. Two milliliter of aqueous extract of dry matter were placed in a 10 cm^3^ volumetric flask, then added 5 mL of distilled water followed by 0.6 mL of 5% sodium nitrite solution. After 5 min, 0.6 mL of AlCl_3_ solution (10%) was added. After another 5 min, 2 mL of 1M NaOH were added and the volume made up to 10 mL with distilled H_2_O. The solution was mixed thoroughly and absorbance noted at 510 nm. TF concentrations were expressed as catechin equivalents g/100 g of dry matter.

### 3.8. Total Carotenoid Content

Total carotenoids were estimated following the method of Gao *et al*. [ 66]. Oil solutions in hexane (1 g/100 mL) were read at 460 nm on a spectrophotometer (U-2001, Hitachi Instruments Inc. Tokyo, Japan). Quantification of amounts was based on the carotene standards and amount of carotenoids was expressed in μg g^−1^ of oil.

### 3.9. DPPH Radical Scavenging Activity

The free DPPH radical scavenging activity of the oil extracts was estimated following the method as described by Sultana *et al*. [ 67] and Iqbal and Bhanger [ 68] with some modifications. Five ml of a freshly prepared solution of 1,1′-diphenyl-2-picrylhydrazyl (DPPH) at concentration 0.025 g/L were added to 1.0 mL of the extract of dry material in methanol. The mixture was incubated at room temperature in the dark for 30 min. Then the decrease in absorbance was measured against a blank at 515 nm (the change in color from deep-violet to light-yellow). Following formula was used to calculate the radical scavenging activity:

(1) DPPH radical scavenging (%)=[(AB-AA)/AB]×100

Where AB is absorbance of blank sample (t = 0 min); AA is absorbance of tested extract solution after 15 min of incubation.

### 3.10. Statistical Analysis

Data for each attribute was subjected to CoStat Computer Program (version 6.303, PMB 320, Monterey, CA, USA) for calculating ANOVA. Mean values were compared with LSD, worked out following Steel and Torrie [ 69].

## 4. Conclusions

From the results of the present analyses, it could be concluded that imposition of drought stress significantly affected the chemical composition of maize kernels and kernel oil, but exogenous application of proline ameliorated the adverse effects of drought on seed chemical composition. These effects of exogenously applied proline on improving seed and seed oil composition were not only exhibited under water deficit conditions but also under non-stressed conditions. Amongst the different studied maize kernel oil characteristics, oil yield, fatty acid composition, oxidative stability and oil phenolics were found to be more vulnerable to drought and positively affected by the foliar application of proline. The exogenous application of proline can thus be effectively used to improve seed and seed oil nutritional quality of maize crop grown not only under water deficit conditions but also under well watered conditions. However, a comparatively more increasing effect was observed in these antioxidative compounds due to the foliar application of proline; under water deficit conditions such as those compared with non-stress conditions, the ameliorating effect of foliar-applied proline was indicated on seed nutritional quality under water deficit conditions.

## Figures and Tables

**Table 1:** Seed proximate composition of two maize (*Zea mays* L.) cultivars as influenced by foliar-applied proline under water stress and non-stress conditions.

Cultivar	Drought treatment	Treatments	Seed oil content	Protein content	Starch content	Sugar content	Ash content	Fiber content	Moisture content
			% of dry weight						

EV-1098	Control	NS	3.87 ± 0.13 ^b^	7.92 ± 0.49 ^a,b^	56.67 ± 8.30 ^c^	1.60 ± 0.16 ^c^	1.87 ± 0.24 ^c^	9.08 ± 0.30 ^e^	9.07 ± 0.67 ^b^
		WS	3.87 ± 0.18 ^b^	7.87 ± 0.35 ^a,b,c^	56.67 ± 5.88 ^c^	1.80 ± 0.02 ^b^	1.85 ± 0.12 ^c^	9.52 ± 0.71 ^d,e^	8.95 ± 1.02 ^b^
		Pro	4.12 ± 0.15 ^a^	8.01 ± 0.73 ^a^	61.33 ± 7.88 ^a,b,c^	2.21 ± 0.16 ^a^	1.97 ± 0.22 ^c^	10.10 ± 0.80 ^b,c^	10.18 ± 0.49 ^a^

	Drought	NS	2.95 ± 0.15 ^d^	6.26 ± 0.67 ^e^	60.33 ± 2.43 ^b,c^	1.13 ± 0.02 ^d^	2.23 ± 0.18 ^b^	10.45 ± 0.82 ^b,c^	5.71 ± 0.18 ^d^
		WS	2.87 ± 0.13 ^d^	6.85 ± 0.75 ^d,e^	59.67 ± 4.44 ^b,c^	1.14 ± 0.01 ^d^	2.29 ± 0.31 ^b^	10.55 ± 0.53 ^b,c^	5.70 ± 0.68 ^d^
		Pro	3.63 ± 0.15 ^c^	7.23 ± 0.68 ^b,c,d^	65.67 ± 6.30 ^a,b^	1.18 ± 0.02 ^d^	2.55 ± 0.24 ^a^	12.12 ± 0.77 ^a^	7.18 ± 0.43 ^c^

Agaiti- 2002	Control	NS	3.72 ± 0.15 ^b,c^	7.59 ± 0.62 ^a,b,c^	60.33 ± 6.12 ^b,c^	1.63 ± 0.08 ^c^	1.77 ± 0.16 ^c^	8.62 ± 0.81 ^f^	8.57 ± 0.29 ^b^
		WS	3.65 ± 0.13 ^c^	7.51 ± 0.90 ^a,b,c^	61.33 ± 9.58 ^a,b^	1.80 ± 0.02 ^b^	1.80 ± 0.10 ^c^	8.65 ± 0.45 ^f^	9.10 ± 0.92 ^b^
		Pro	4.16 ± 0.14 ^a^	8.01 ± 0.66 ^a^	68.00 ± 7.22 ^a^	2.18 ± 0.16 ^a^	1.95 ± 0.15 ^c^	9.89 ± 0.30 ^c,d^	10.24 ± 0.51 ^a^

	Drought	NS	2.36 ± 0.08 ^e^	6.59 ± 0.48 ^d,e^	63.67 ± 3.41 ^a,b^	1.14 ± 0.01 ^d^	2.27 ± 0.23 ^b^	10.65 ± 0.57 ^b^	5.35 ± 0.31 ^d^
		WS	2.42 ± 0.09 ^e^	6.76 ± 0.81 ^d,e^	61.67 ± 9.59 ^a,b^	1.14 ± 0.01 ^d^	2.23 ± 0.18 ^b^	10.54 ± 0.46 ^b,c^	5.46 ± 0.13 ^d^
		Pro	2.82 ± 0.12 ^d^	7.18 ± 0.54 ^c,d^	68.33 ± 3.81 ^a^	1.16 ± 0.01 ^d^	2.45 ± 0.28 ^a,b^	12.27 ± 0.86 ^a^	6.95 ± 0.31 ^c^

LSD at 5%			0.15	0.73	7.40	0.10	0.23	0.72	0.63

Data are presented as means ± standard error. Means within the same column sharing the same superscript letters do not differ significantly at the 5% level. NS: No spray; WS: Water spray; Pro: 30 m*M* proline.

**Table 2:** Seed oil physico-chemical attributes of two maize (*Zea mays* L.) cultivars as influenced by foliar-applied proline under water stress and non-stress conditions.

Cultivar	Drought treatment	Treatments	Saponification value (mg of KOH g^−1^ of oil)	Un-saponifiable matter (%)	Iodine value (g of I 100g^−1^ of oil)	FFA (mg of KOH/g of oil)	Peroxide value (meq kg^−1^)	*p*-anisidine value	Dienes [ɛ_1 cm_(λ_232 nm_)]	Trienes [ɛ_1 cm_(λ_268 nm_)]
EV-1098	Control	NS	196.12 ± 10.78 ^a^	2.11 ± 0.23 ^d,e^	115.16 ± 5.53 ^a,b^	1.41 ± 0.08 ^c^	6.30 ± 0.56 ^f^	1.87 ± 0.057 ^f^	1.83 ± 0.005 ^b^	1.54 ± 0.009 ^e^
		WS	193.93 ± 07.42 ^a,c^	2.25 ± 0.09 ^b,c,d^	114.37 ± 4.96 ^b^	1.42 ± 0.08 ^c^	6.41 ± 0.47 ^d^	1.89 ± 0.066 ^f^	1.79 ± 0.005 ^d^	1.64 ± 0.005 ^d^
		Pro	201.67 ± 10.31 ^a^	1.97 ± 0.04 ^e^	121.56 ± 8.05 ^a^	1.29 ± 0.05 ^d^	5.58 ± 0.47 ^h^	2.12 ± 0.106 ^d,e^	1.63 ± 0.006 ^h^	1.63 ± 0.007 ^d^

	Drought	NS	178.55 ± 09.14 ^b^	2.35 ± 0.19 ^a,b^	115.34 ± 3.90 ^a,b^	1.70 ± 0.11 ^a,b^	7.60 ± 0.37 ^a^	2.14 ± 0.072 ^c,d,e^	1.71 ± 0.008 ^e^	1.81 ± 0.006 ^b^
		WS	179.74 ± 10.28 ^b^	2.28 ± 0.16 ^b,c^	112.95 ± 7.37 ^c^	1.76 ± 0.08 ^a^	7.56 ± 0.44 ^a^	2.14 ± 0.057 ^c,d^	1.69 ± 0.007 ^f^	1.68 ± 0.006 ^c^
		Pro	188.08 ± 12.93 ^b,c^	2.14 ± 0.09 ^c^	121.86 ± 7.19 ^a^	1.48 ± 0.10 ^c^	6.34 ± 0.42 ^e,f^	2.24 ± 0.097 ^b,c^	1.60 ± 0.008 ^i^	1.55 ± 0.008 ^e^

Agaiti- 2002	Control	NS	191.07 ± 11.27 ^a^	2.11 ± 0.18 ^d,e^	113.49 ± 3.40 ^c^	1.35 ± 0.07 ^d^	6.15 ± 0.37 ^g^	2.09 ± 0.091 ^d,e^	1.88 ± 0.011 ^a^	1.68 ± 0.005 ^c^
		WS	189.26 ± 11.50 ^b,c^	2.16 ± 0.15 ^c^	113.75 ± 4.28 ^c^	1.40 ± 0.06 ^c^	6.37 ± 0.43 ^d,e^	2.04 ± 0.189 ^e^	1.81 ± 0.009 ^c^	1.64 ± 0.005 ^d^
		Pro	198.67 ± 07.27 ^a^	1.95 ± 0.11 ^e^	115.68 ± 5.28 ^a,b^	1.28 ± 0.04 ^d^	5.50 ± 0.56 ^i^	2.10 ± 0.068 ^d,e^	1.66 ± 0.011 ^g^	1.55 ± 0.004 ^e^

	Drought	NS	175.38 ± 11.03 ^b^	2.47 ± 0.14 ^a^	116.60 ± 5.41 ^a,b^	1.64 ± 0.08 ^b^	6.59 ± 0.39 ^c^	2.33 ± 0.091 ^a,b^	1.71 ± 0.009 ^e^	1.87 ± 0.006 ^a^
		WS	179.86 ± 03.80 ^b^	2.30 ± 0.11 ^b^	113.83 ± 6.29 ^c^	1.64 ± 0.08 ^b^	6.79 ± 0.29 ^b^	2.32 ± 0.040 ^a,b^	1.64 ± 0.009 ^h^	1.82 ± 0.010 ^b^
		Pro	186.14 ± 10.24 ^b,c^	2.15 ± 0.15 ^c^	116.64 ± 7.55 ^a^	1.46 ± 0.08 ^c^	6.33 ± 0.57 ^e,f^	2.42 ± 0.070 ^a^	1.55 ± 0.008 ^j^	1.55 ± 0.005 ^e^

LSD at 5%			11.07	0.16	6.62	0.09	0.05	0.01	0.10	0.01

Data are presented as means ± standard error. Means within the same column sharing the same superscript letters do not differ significantly at the 5% level. NS: No spray; WS: Water spray; Pro: 30 mM proline; FFA= Free fatty acids.
